# Implementation of an Audience Response System in a Case Conference Curriculum: Results of a Placebo-Controlled Trial

**DOI:** 10.7759/cureus.13512

**Published:** 2021-02-23

**Authors:** Ghulam Rehman Mohyuddin, Katherine Lester, Laura Thomas, Leigh M Eck, Jessica R Newman

**Affiliations:** 1 Internal Medicine Medical Education, University of Kansas Hospital & Medical Center, Kansas City, USA; 2 Pulmonology/Critical Care, University of Kansas Hospital & Medical Center, Kansas City, USA; 3 Infectious Diseases, University of Kansas Hospital & Medical Center, Kansas City, USA

**Keywords:** audience response system, medical education, case conference, curriculum development

## Abstract

Audience response systems engage learners and facilitate the assimilation of the material. We assessed whether incorporation of one system into a resident case conference would increase retention of information and attentiveness. Pre-tests were administered prior to case conferences. The University Hospital incorporated Poll Everywhere into a conference and the Veterans Administration hospital did not. Participants rated self-perceived attentiveness and completed a post-test following conference. There was an increase in post-test scores compared to pre-tests. There was no significant difference in self-perceived attentiveness or post-test scores between sites. The use of audience response did not increase retention of material or perceived attentiveness when incorporated into the conference.

## Introduction

Didactics covering core specialty concepts are integral to graduate medical education. Time devoted to resident didactic education is increasingly constrained due to mounting clinical responsibilities, documentation requirements, and duty hour restrictions [1]. It is imperative that objectives presented during didactics are retained by residents to increase preparation for board examinations and to work towards the achievement of professional competency [2]. Resident case conferences are commonly incorporated into internal medicine (IM) didactics to build skills in clinical reasoning [3]. During instructional activities, a resident typically introduces a teaching case, reviews a differential diagnosis, and offers salient teaching points.

Audience response systems (ARS) have become increasingly available and are well received by learners [4-6]. Previous studies have shown that the addition of ARS to didactics enhances learning and retention [7-9]. Quiz scores following traditional family medicine lectures were noticeably poor compared to lectures that included ARS [7]. An additional study found the use of ARS increased comprehension and retention of the information presented at radiology case conferences [8]. The incorporation of ARS into IM residency didactics, specifically case conferences, has not been well studied. Our objective was to assess whether the integration of ARS into IM resident case conferences would increase retention of information and self-perception of attentiveness.

## Materials and methods

This study primarily included IM residents, but also medical students rotating through a university-affiliated IM residency training program. Resident case conferences occurred on average once weekly as part of the midday core didactic series during the 2017-2018 academic year. Ten case conferences given over a three-month period were included in the study.

The web-based ARS, Poll Everywhere (www.PollEverywhere.com), was chosen given its broad capabilities and free smart-phone application. Participants were able to respond via app, on the web or by SMS text. Poll Everywhere allows audience members to respond in real-time to questions posed by the presenter. The participant's answers can then be displayed on the screen. Options for questions include open-ended, ranking, word cloud, graphic selection or multiple-choice, with a running tally of responses shown on the screen.

Two sites were included in the study, the university hospital (UH) where Poll Everywhere was incorporated into case conferences, and the Veterans Administration (VA) hospital where Poll Everywhere was not incorporated. At the UH, three interactive Poll Everywhere questions related to the case designed to engage the audience members were added to the PowerPoint slide deck for each conference (PowerPoint, Microsoft).

A five-item test, created by one investigator, was administered at the beginning of and conclusion of the conference at both sites. The test included three multiple-choice questions and two open-ended questions that were related to the conference subject content. The same case subject and pre- and post-tests were utilized at both sites. To ensure the content of the tests was included in the session, chief residents facilitated the case discussion and ensured uniformity of content coverage. At the conclusion of the post-test, participants rated self-perceived attentiveness using a five-point Likert item scale. Results from the tests were imported into statistical software SPSS version 23 (SPSS, Inc., Chicago, IL, USA) for data analysis. Data values were presented as mean, median, and standard deviation.

Participants were provided with an electronic letter describing the study and its volunteer nature prior to and at the time of each conference. The tests were collected anonymously. This educational study was reviewed by the University of Kansas Medical Center Human Subjects Committee and deemed not human subjects research.

## Results

A total of 183 tests were completed from a total of 270 attendees over the study time period (completion rate=67.78%). At both sites, a slightly higher number of post-tests were collected than pre-tests. A similar quantity of pre-tests was collected at UH (n=93, mean 2.44, median 2.00, standard deviation 1.22) and the VA hospital (n=90, mean 2.27, median 2.00, standard deviation 1.15) with no statistically significant difference (p=0.334) between scores.

Similarly, there was no statistically significant difference (p=0.444) between post-test scores at UH (n=124, mean 3.36, median 3.00, standard deviation 1.11) and the VA hospital (n=118, mean 3.48, median 4.00, standard deviation 1.32). At both sites, there was a significant difference in pre- and post-test scores (p=0.0001). Self-perceived attentiveness was rated highly at UH (n=115, mean 4.07, median 4, standard deviation 0.72) and the VA hospital (n=89, mean 4.07, median 4, standard deviation 0.77). However, there was no statistically significant difference between self-perceived attentiveness at either site (p=0.568).

## Discussion

Medical knowledge, achieved through various platforms of graduate medical education, is a core competency defined by the Accreditation Council for Graduate Medical Education (ACGME) [10]. However, with escalating clinical documentation responsibilities and duty hour restrictions, residents have reported a decline in time devoted to medical education [1,2,11]. This is despite a demand to acquire an escalating body of specialty-related knowledge and clinical practice skills with a decline in protected time for study [12]. Therefore, optimal and efficient learning strategies need to be evaluated to ensure residents are attentive and retain material presented to them in the conference.

Incorporating ARS into lectures allows audiences to be engaged, which in theory can increase attentiveness [13-15]. Additionally, these systems allow the presenter to explore the audience’s comprehension during the presentation, which can increase a learner’s retention by clarifying material and providing immediate feedback [16]. However, in this study, we did not identify a statistical difference in retention when incorporating an ARS into IM case conferences. Furthermore, there was no difference in self-perceived attentiveness at either site.

Limitations to our study include the small sample size at a single IM residency program. In addition, there were different rates of attendance. There were more post-tests than pre-tests collected at each site, which was likely accounted for by late arrivals to conferences. It is possible that with larger sample size and equal pre- and post-test completion rates that the power to detect a statistical significance in retention and self-perceived attentiveness would be increased. Another consideration was that conference presenters at the two sites were different, introducing distinctive speaking styles and slide sets, though standardization was attempted by having chief residents facilitate to ensure relevant content was being covered in the discussion.

Differences intrinsic to the conference site (UH versus VA) such as the size of the learning group, conference room climate/seating could also have affected the results of the study. Furthermore, it is unknown whether incorporating ARS into a less inherently active learning format (such as a traditional lecture) would lead to improved retention and attentiveness. Our results were at a population level with anonymous tests collected, and thus individual learner pre- and post-test scores could not be compared.

## Conclusions

In conclusion, the use of an ARS, Poll Everywhere, did not increase retention of material or self-perceived attentiveness when incorporated into resident case conference. Given learner acceptance and enjoyment of this method, conducting a similar study on a larger scale may be useful and further studies on the incorporation of tools such as ARS into differing pedagogies of residency didactic sessions are needed. Other methods to enhance learner attentiveness and retention during residency didactic conferences must also be explored.
